# Soil flushing of cresols contaminated soil: application of nonionic and ionic surfactants under different pH and concentrations

**DOI:** 10.1186/s40201-014-0129-z

**Published:** 2014-11-11

**Authors:** Saeid Gitipour, Khadijeh Narenjkar, Emad Sanati Farvash, Hossein Asghari

**Affiliations:** Graduate Faculty of Environmant, University of Tehran, Tehran, Iran

**Keywords:** Contaminated soil, Cresols, SDS, Soil flushing, Surfactants, Triton X-100

## Abstract

In this study, the viability of soil flushing on the removal of cresols (meta-, ortho-, and para-cresols) from contaminated soil has been investigated. High production and distribution of cresols in the environment indicate their potential for a widespread exposure to humans. The presence of these compounds in soil could cause a significant threat to environment, as they are toxic and refractory in nature. Cresols are persistent chemicals which are classified by the United State Environmental Protection Agency (U.S.EPA) as Group C, possible human carcinogens. Soil flushing is one of the soil remediation technologies which could by applied for treatment of hydrocarbon contaminated soil. Flushing of the contaminated soil samples was carried out by using sodium dodecyl sulfate (SDS) and Triton X-100 surfactant solutions at the concentrations of 0.1%, 0.2%, 0.3%, and 0.4% (W/W). Three acidic, neutral, and alkaline environments were utilized by adjusting pH of the washing solutions at 3, 7 and 12 to evaluate the effect of washing environment in removing cresols. The results of this research denote that the highest removal efficiencies of 79.6% and 83.51% were achieved for m-cresol and total o- and p-cresols, respectively, under the alkaline environment of pH12 at 0.4% (W/W) SDS concentration. Regarding performance of Triton X-100, the removal efficiencies of 80.26% and 80.14% for the above cresols were attained under similar conditions. Hence, illustrating the effectiveness of surfactants in soil flushing remediation of cresols contaminated soil.

## Background

In recent years, contamination generated by petroleum compounds has raised concern all over the world [1]. Petroleum pollution is a global disaster that is a common phenomenon in the oil-bearing and industrial regions [2]. Because of the low solubility of hydrophobic organic compounds (HOCs) in water, the residual organic phase usually represents a long-term contamination source for soil and groundwater [3]. Cresols are isomeric substituted phenols with a methyl substituent at either ortho, meta or para position relative to the hydroxyl group [4]. These compounds are considered to be toxic and have been classified as hazardous pollutants [5]. EPA has classified cresols (m-, o-, and p-cresol) as Group C, possible human carcinogens [6-8].

In the recent decades, a broad range of physical, chemical, and biological methods have been applied for the remediation of soil contaminated with hydrophobic organic compounds [9]. Among all remediation technologies for treating hydrophobic organic contaminated soil, flushing is an effective and economical method, where a flushing fluid is applied to the surface of the contaminated site or injected into the saturated contaminated zone [10]. Soil flushing is the extraction of contaminants from the soil with water or other suitable aqueous solution [11]. The method usually works by applying water to the soil. Pollutants dissolved in the flushing solution are leached into the groundwater and then extracted [12,13].

Researchers have studied the usefulness of surfactants for the recovery of soils and aquifers polluted by HOCs [14-16]. As water solubility is the controlling mechanism of dissolution, additives (surfactants, cosolvents, etc.) are used to enhance efficiencies and reduce the treatment time compared to the use of water alone [17]. There are different types of surfactants, including ionic surfactant (e.g., sodium dodecyl sulfate or SDS), non-ionic surfactant (e.g., Triton X-100) and zwitterionic surfactant (e.g., CHAPS) [18]. Anionic and nonionic surfactants have usually been chosen in surfactant remediation technology [19,20].

The aim of this research was to evaluate the efficiencies of SDS, Triton X-100, and water in desorption of cresols from contaminated soil, and to assess the effects of pH, and washing solutions concentrations on the viability of soil flushing treatment technology.

## Materials and methods

### Materials

In this research, a poorly-graded sandy soil was used for evaluation of the efficiency of flushing experiments. Some major engineering characteristics of the soil are presented in Table 1.

**Table 1:** **Soil characteristics**

**Property**	**Test method**	**Value**
Water content	ASTM D 2216	1.83%
Specific gravity	ASTM D 854-92	2.67
pH	ASTM D 4972	7.46
Electrical conductivity	ASTM D 2974	213 (μs cm^−1^)
Organic content	ASTM D 2974	1.48%
USCS classification system	ASTM D 422-63	poorly-graded sand
Porosity (n)	―	0.46

Triton X-100 (TX-100) is a nonionic surfactant which has a hydrophilic head on one side and a hydrophobic tail on another side [21]. They are extensively used as preservatives or antiseptic agents in industrial and commercial products [22]. The micro heterogeneous environment of SDS contains a negatively charged surface and a hydrophobic interior [23]. For this study, SDS and Triton X-100 were purchased from Merck and Scharlau Chemie, respectively. Selected physiochemical characteristics of the surfactants used throughout this research are presented in Table 2.

**Table 2:** **Selected physicochemical properties of surfactants in this study**

**Characteristic**	**SDS**	**Triton X-100**
Molecular formula	C_12_H_25_OSO_3_Na	C_14_H_22_O(C_2_H_4_O)_10_
Chemical structure		
Molecular weight (g mol^−1^)	288.38	625
CMC (mM l^−1^)	3.32-8.4	0.2-0.31
Purity (%)	90 ~	100-98
specific gravity	1.1 (20°C)	1.064-1.067 (20°C)
pH	6.0-9.0 (Water 1%)	6.0-8.0 (Water 1%)

Cresols, monomethyl derivatives of phenol are produced commercially by chemical synthesis or by distillation from petroleum or coal tar [24]. Physically, they are white crystalline solids or yellowish liquids with a strong phenol-like odor. The compounds are highly flammable, moderately soluble in water and soluble in ethanol, ether, acetone, or alkali hydroxides [25]. O-cresols are used as solvents, disinfectants and chemical intermediate, while pcresol is utilized in the formulation of antioxidants, fragrance and dye industries [26] and m-cresol is used to produce certain herbicides, to produce antioxidants, and to manufacture the explosive, 2,4,6-nitro-m-cresol [27]. Three isomers of cresols including meta-, ortho-, and para-cresol were purchased from Merck Chemical Company.

### Soil flushing remediation experiments

**Figure 1:**
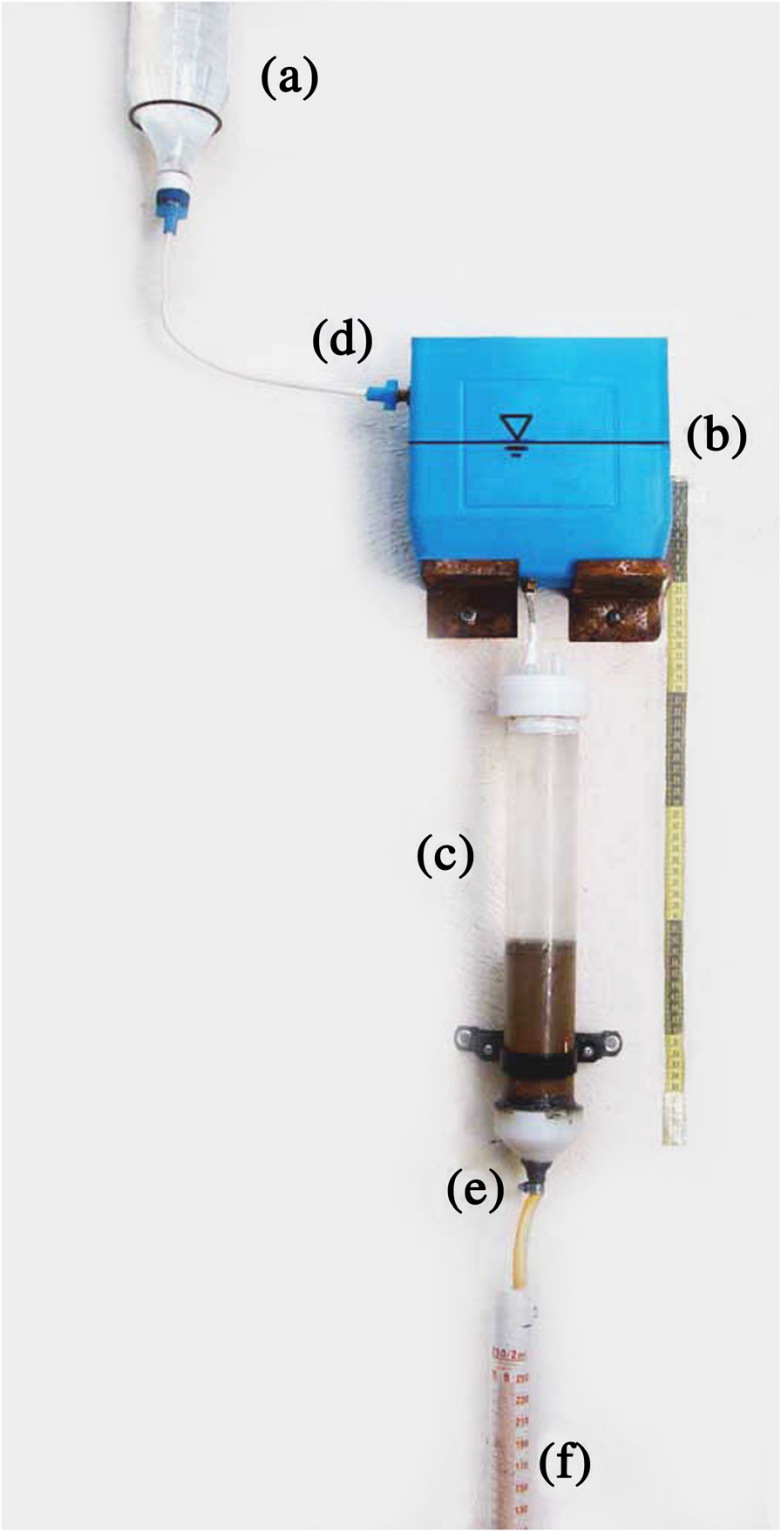
**Soil flushing apparatus, (a) primary surfactant solution reservoirs, (b) secondary surfactant solution reservoirs, (c) glass cylindrical column (d) surfactant inflow valve, (e) leachate outflow valve, and (f) graduated cylinder.**

The flushing apparatus used in the research consisted of a glass cylindrical column (internal diameter of 5 cm and the height of 35 cm), primary and secondary surfactant solution reservoirs, and surfactant solution and leachate control valves. Figure 1 presents the soil flushing equipment used throughout the experiments.

### Methods

Table 3 presents the permissible concentrations of different cresol isomers in residential soil regulated by various state department environmental qualities.

**Table 3:** **Permissible cresols concentrations in soil based on various environmental departments**

**Concentrations in residential soil (mg Kg** ^**−1**^ **)**		**Environmental department**
**para-cresol**	**meta- and ortha-cresol**	
310	3100	Arizona^a^
310	3100	Wyoming^b^
300	2900	Florida^c^
393	2675	EPA^d^

^a^Arizona Department of Environmental Quality [28].

^b^Wyoming Department of Environmental Quality [29].

^c^Florida Department of Environmental Protection [30].

^d^EPA Cleanup Criteria [31].

Based on the cleanup levels stated in Table 3, o-, m-, and p-cresols concentrations in the samples were set at 4000 ppm, 4000 ppm, and 1000 ppm, respectively. Next, samples weighing 500gr each, were uniformly spiked with different cresol isomers (o-cresol and m-cresol each 2gr, and p-cresol 0.5gr) to bring their concentrations to 9000 ppm in the soil. To accomplish the uniformity, cresols were diluted in dichloromethane, added to the soil, and thoroughly blended to obtain a homogenous mix. Following spiking the specimens, the glass cylindrical column was utilized and the soil was placed in it at three different layers. A rubber mallet was used for compacting each layer to a height of 5 cm (total of 15 cm of soil column). SDS and Triton X-100 surfactants at solution concentration of 0.1%, 0.2%, 0.3% and 0.4% (W/W) were used to flush the soil specimens. Total of 8 pore volumes of the surfactant solutions was used to flush the samples. Flushing test were carried out by using surfactant solutions at different pH values of 3, 7, and 12 to evaluate the effect of solution environment on removal of cresols from the samples. Following the experiments, the leachates collected from each soil flushing experiment were passed through a 0.45 μm membrane filter to remove suspended solids and then was transferred into 25 mL screw-capped vials. The filters were then washed with a methanol solution to remove any cresol residues which possibly might have been remained on their surfaces. Next the vials were stored in a refrigerator at 4°C for High-performance liquid chromatography (HPLC) analysis. HPLC apparatus was used for analysis of the samples. The cresols analysis were accomplished in accordance with U.S.EPA SW-846 and Method 8270 [32].

### HLPC Sample analyzes

Agilent 1100 HPLC system was used for analyses of the cresol contaminants in soil samples. The analysis was carried out by employing a modular Shimadzu LC-10 system comprised of a LC-10 AD pump, a CTO-10A column oven, a SPD-10A UV-DAD detector with wavelength of 274 nm, a FLD detector, a CBM-10A interface, and a LC-10 Workstation. A LC-18 column (250 mm × 4 mm ID × 5 mm) was employed at 26°C and separations were conducted in the isocratic mode, using acetonitrile:acetate buffer (30:70; v/v) at a flow rate of 0.3 mL min − 1, with an injection volume (“loop”) of 20 mL and an accuracy of ±2%. The concentration of acetate buffer was 266 mM (101 mM of acetic acid and 165 mM of Sodium acetate trihydrate) in the HPLC analyses.

## Results and discussion

### Removal efficiency of cresol isomers

**Figure 2:**
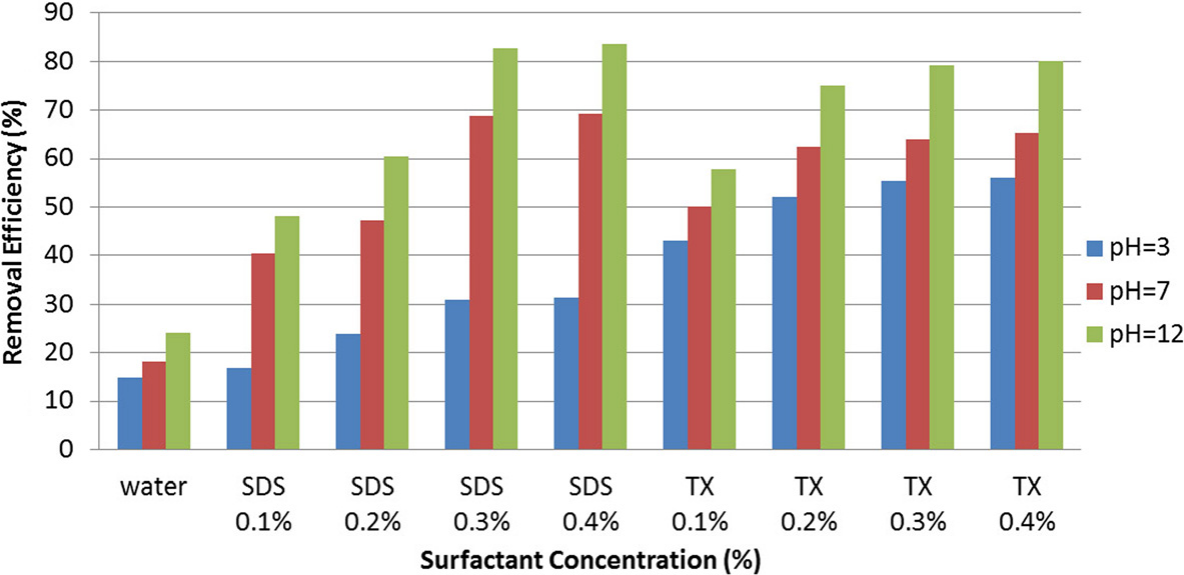
**Total ortha- and para-cresols removal efficiencies exposed to various washing solutions under different pH conditions and concentrations.**

**Figure 3:**
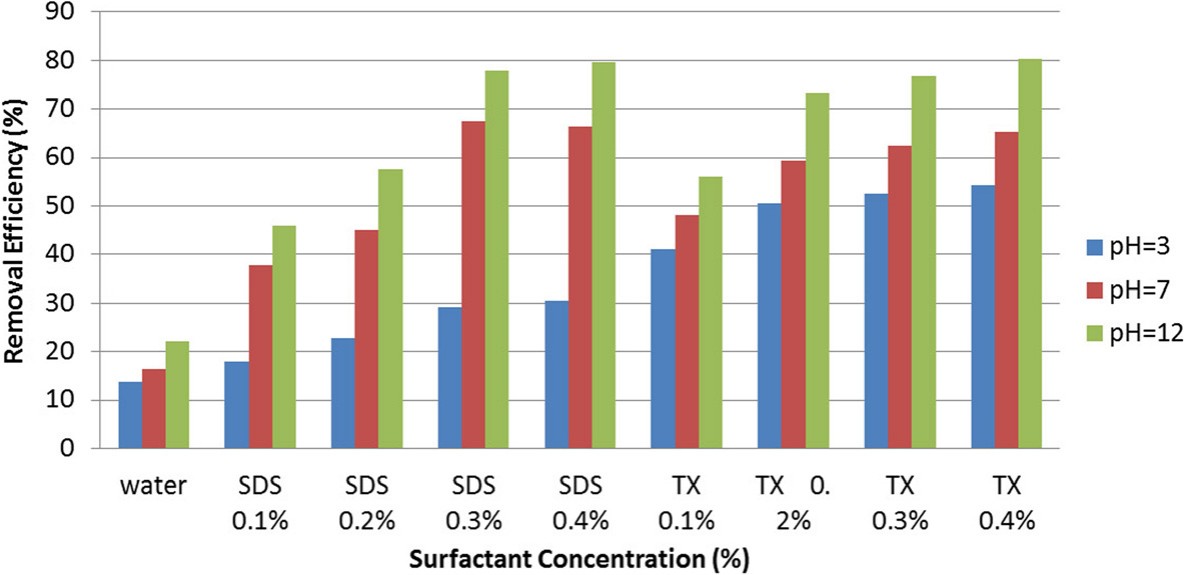
**Meta-cresol removal efficiencies exposed to various washing solutions under different pH conditions concentrations.**

In this study, removal efficiencies of cresols from contaminated soil under various surfactant concentrations and pH values were scrutinized. Due to the overlap of o- and p-cresols’ peaks during the HPLC analysis with Agilent 1100 HPLC system, these contaminants could not be differentiated from each other throughout the analysis, hence, their total concentrations have been regarded in this research. Figures 2 and 3 present the results of removal efficiency values for total o- and p-cresols, and m-cresol under the application of SDS, Triton X-100, and water solutions.

As illustrated in Figure 2, the removal efficiencies of total o- and p-cresols flushed with water, SDS, and Triton X-100 solutions varied from 14.9% to 24%, 16.8% to 83.5%, and 43.1% to 80.1%, respectively. For m-cresol, the removal efficiencies under the above solutions presented similar trend of removals as total o- and p-cresols (see Figure 3). The highest removal efficiency of m-cresol was 22%, 79.6%, and 80.26% using water, SDS, and Triton X-100, respectively.

The figures also demonstrate that the removals of cresol isomers have direct relationship with surfactant type and concentration, and pH of flushing solution. The effects of aforementioned testing parameters on cresols removals are discussed below.

### Statistical analysis

Statistically significant differences between cresol isomers removals with surfactants were evaluated by an analysis of Variance using the Minitab 16 program. The p values for both SDS and Triton-X 100 surfactants at different pH values and concentrations were obtained to be less than 0.01 showing a significant difference between the above parameters. The regression analysis conducted on the removal efficiency values of surfactants versus their concentrations and pH values are presented in Table 4.

**Table 4:** **Removal efficiencies values according to regression analysis**

**Contaminant**	**Surfactant type**	**Regression equation**
Ortha and Para cresol	SDS	RE = −6.8 + 110.5 SDSC +3.9 pH
Ortha and Para cresol	Triton-X 100	RE = 15.3 + 112.0 TXC +2.1 pH
Metha cresol	SDS	RE = 5.5 + 103.9 SDSC + 3.7 pH
Metha cresol	Triton-X 100	RE = 13.8 + 110.7 TXC + 2.1 pH

*Abbreviation*: RE = Removal Efficiency; SDSC = SDS Concentration; TXC = Triton-X 100 Concentration.

**Figure 4:**
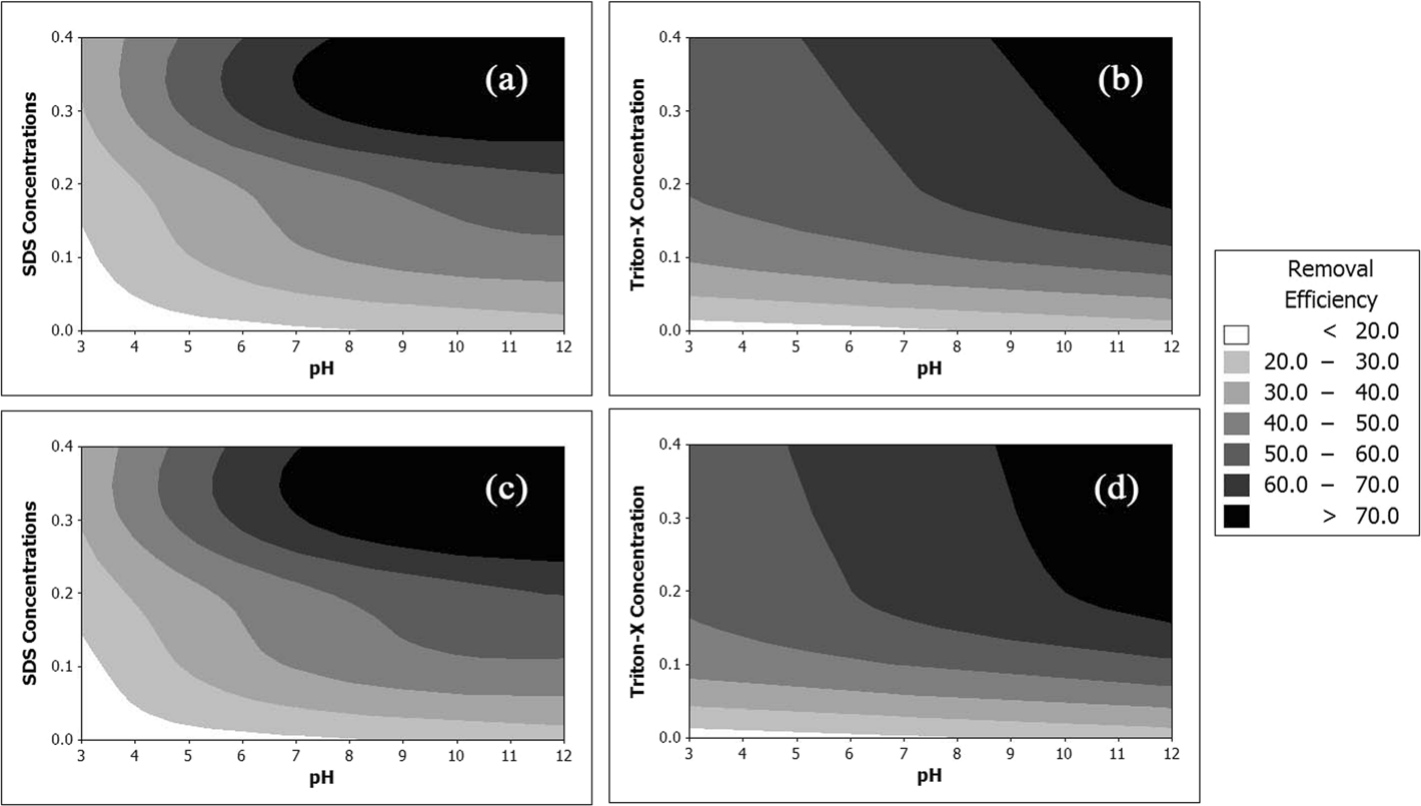
**Contour lines of cresols’ removal efficiencies versus pH and surfactants concentrations for (a) m-cresol using SDS, (b) m-cresol using Triton-x 100, (c) o and p-cresol using SDS, and (d) o and p-cresol using Triton-X 100.**

Based on the regression equations presented in Table 4, the contour lines of cresols’ removal efficiency values versus pHs and concentrations of surfactants are presented in Figure 4.

### Surfactant type versus cresols removal efficiencies

The results of analysis denote that the removal efficiencies of cresol isomers (m-, o-, and p-cresols) flushed with SDS and Triton X-100 surfactants were significantly higher than that of water, indicating that water was the least effective solution in flushing of the soil. This could be attributed to surfactants amphiphilic molecules that reduce aqueous surface tension and increase the solubility of hydrophobic organic compounds [33]. Furthermore, the higher average removal efficiencies of cresols with Triton X-100 could be attributed to superior micelle formation that increased the solubility of pollutants in Triton X-100 solutions. Similar information was reported by Muherei and Junin, indicating that nonionic surfactants (i.e. Triton X-100) due to their lower CMC values perform more efficiently in removing organics than ionic surfactant (i.e. SDS) [34] (see Table 2).

### Effect of surfactant concentration on cresols removal efficiency

As shown in Figures 2 and 3, the removal efficiencies of contaminants in the samples were increased with increasing of SDS and Triton X-100 concentrations up to about 3% of the surfactants. However, further increase in concentrations of surfactants to 4% did not appear to have any significant increases in removals of the cresols from the soil samples. Indicating the optimum soil flushing of contaminants at about 3% of surfactant concentrations. The figures also illustrate that, in the range of 1% to 3% concentration of surfactants, on average Triton X-100 removed the contaminants more effectively as compared with SDS.

### Effect of solution pH on cresols removal efficiencies

The effect of the solution pH on removal of cresols was investigated for both of the surfactants and water. As presented in Figures 2 and 3, the removal efficiencies of cresols increased as the pH of surfactant solutions changed from 3 to 12. According to the analysis of soil flushing effluents, the highest removal of cresols occurred at the alkaline environment of pH12. Similar results for pH effects on cresols removals were reported by Evangelista et al. (1990) which indicated the higher cresols removals at the pH11.5 as compared to pH10.5 and pH9.3 washing solutions [35]. Furthermore, Salehian et al. studied the removal efficiency of diesel-contaminated soil using SDS solution as a flushing liquid. They reported that the removal efficiencies of contaminants in alkaline and neutral phases were higher than that of acidic environments [36].

## Conclusions

In the present study, soil flushing tests were conducted to evaluate the effects of surfactants’ concentrations and pH of solutions on removal of cresols from soil. The study results indicate that cresols removals had a close relationship with pH and concentrations of SDS and Triton X-100 surfactants. For both surfactants and water, cresols removals from soil increased with the increase of flushing solutions’ pH. Maximum removal of cresols was achieved under alkaline environment of pH12. Moreover, increase in SDS and Triton X-100 concentrations from 1% to 4% further increased the removal efficiencies of cresols in soil, with the optimum flushing at about 3% of surfactant concentrations. The above findings are in agreement with the results of a study conducted by Rosas et al., indicating that the higher extraction efficiencies of cresols was achieved by using higher concentration of nonionic Tween 80 surfactant [4]. On average, in the test conducted under different pH and concentration conditions, Triton X-100 presented more effective cresols’ removal efficiencies than SDS surfactants. The results also indicated that the overall cresols removals in the tests increased in the order of water < SDS < Triton x-100, illustrating the effectiveness of surfactants in remediation of cresols contaminated soil.
